# Combined Rib-Latissimus Flap; the “Picket Fence” Concept for Reconstruction of Upper Tibia Defects

**DOI:** 10.5812/kowsar.22517464.3553

**Published:** 2012-01-15

**Authors:** Shahram Nazerani, Mohammad Hosein Kalantar Motamedi, Mohamad Reza Ebadi, Adel Ebrahimpoor, Tara Nazerani, Bardia Bidarmaghz

**Affiliations:** 1 Department of Surgery, Tehran University of Medical Sciences, Tehran, IR Iran; 2 Trauma Research Center, Baqiyatallah University of Medical Sciences, and Attending Faculty , Azad University of Medical Sciences, Tehran, IR Iran; 3 Asia Medical Center, Tehran, IR Iran; 4 Shahid Beheshti University of Medical Sciences, Tehran, IR Iran; 5 Tehran University of Medical sciences, Tehran, IR Iran

**Keywords:** Tibia, Reconstructive Surgical Procedures, Ribs

## Abstract

**Background:**

Upper tibia defects ,type3b Gustilo, due to huge size and volume are very difficult to reconstruct; usually several operations are needed for bone and soft tissue defects and the definite one stage reconstruction is yet to be found.

**Objectives:**

In this article we reintroduce the rib- latissimus flap as an acceptable method to reconstruct tibia defects in selected cases.

**Materials and Methods:**

The latissimus muscle with one or two ribs revascualrized by reverse flow from perforators is harvested; the ribs are bisected after harvest yielding four to six struts of vascularized bone to fill the huge upper tibia defect. Internal fixation is very important and we favor LCP plates for long bone fixation and the rib struts are fixed in place by small titanium screws to maintain the “picket fence” design. The muscle is then wrapped around the ribs and the defect is completely reconstructed.

**Results:**

During the past 9 years we have used the rib-latissimus dorsi (RLD) muscle flap, without serratus muscle, in 7 patients with combined bone and soft tissue defects of the upper tibia. All the flaps healed without any major complications and only one stress fracture was seen and treated. The ribs healed and in a median of 14 months hypertrophied to the size of the upper tibia. Nonunion was not observed and patients with lower extremity defects were able to bear full weight within an average of seven months.

**Conclusions:**

The fractures of tibia type 3a and 3b Gustilo are devastating injuries requiring several operations. Several combinations of RLD-Serratus have already been reported but a rib-LD muscle with “picket fence” design has not been reported .The RLD transfer with two ribs divided into four struts for bone coverage and muscle to cover all the upper tibia soft tissue defect can be a useful tool in the armamentarium of the surgeon treating combined defects in a single stage.

## 1. Background

Lower extremity trauma, with open high-energy soft-tissue injuries, is frequently encountered at trauma centers and often requires microsurgical involvement. These wounds are frequently left open and require repeated debridements, resulting in large soft-tissue defects (1-3). Early reports by Buncke in 1977 advocated use of vascularized ribs for limb reconstruction; however it had been done in Japan by Ueba in 1973 who failed to report it; its use shifted from extremity to maxillofacial surgery. The introduction of the free vascularized fibula soon became the workhorse of reconstruction in bone defects. It is however, being replaced by bone transport methods (1, 3). Reconstruction in one stage prevents formation of dense scar tissue inherent to multiple interventions. Additionally, less bone resorption is seen if vascularized bone is used. Therefore, a more undisturbed tissue composition at the end is guaranteed. Moreover, rapid rehabilitation of moving function is possible with improvement in the final result. Finally, morbidity is lowered by using a single donor site, and costs are minimized. We present seven cases in which the fifth and seventh rib including the latissimus dorsi muscle and a paddle of skin have been used to reconstruct huge upper tibia defects. The Gustilo classification, the most widely accepted method of categorizing open fractures, defines three grades. Grade IIIB and C injuries have extensive tissue damage with periosteal stripping, making local soft-tissue coverage unfeasible; grade IIIC injuries have vascular injuries requiring repair. Many centers began to reconstruct the majority of grade III fractures with free-tissue transfer. However, free flaps represent the highest rung on the reconstructive ladder, requiring technically demanding, costly, and time-consuming operations, with significant complication rates, donor-site morbidity, and failure rates (4, 5). Today, many feel that the best method irrelevant to technical sophistication should be used primarily (6, 7). The RLD flap with the rib/s vascularized on the secondary segmental arteries of muscle in a retrograde fashion was used in 7 patients and in all cases a complete coverage and a high degree of function was obtained with a good cosmesis. This flap has the ability of completely cover the bisected ribs. The “picket fence” concept implies using one rib bisected into two segments and placed side by side with an LD flap to fill huge defects of the upper tibia (8-11). Previous studies have not reported the bisecting of the rib and bone filling as a one stage procedure for upper tibia defects.

## 2. Objectives

In this article we reintroduce the rib- latissimus flap as an acceptable method to reconstruct tibia defects in selected cases.

## 3. Materials and Methods

**Figure 1.**
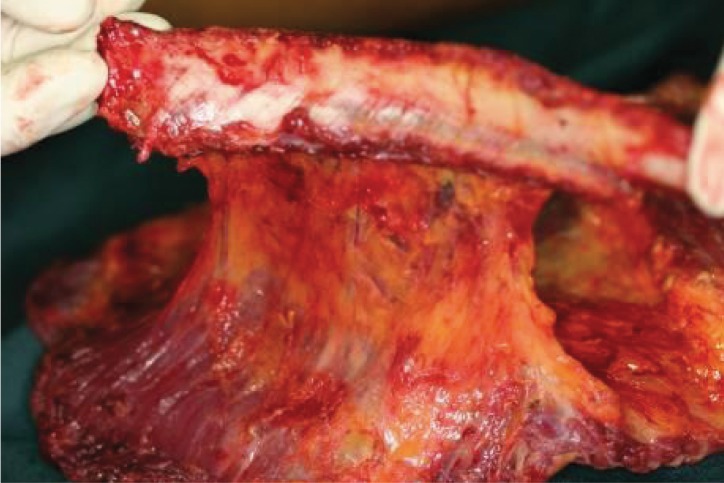
The harvested RLD flap, notice the perforators which normally go into the muscle now nourish the rib through a reverse flow from the muscle.

**Figure 2.**
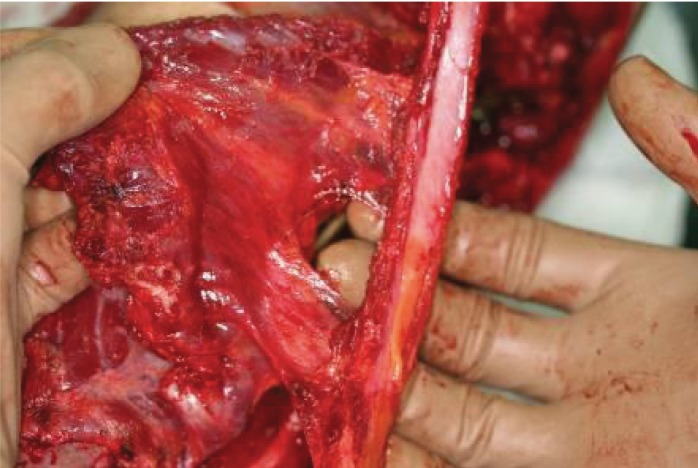
The donor site of the rib and LD muscle harvest is seen with a remaining rib maintaining the contour of the thoracic cavity: and the serratus muscle covers the upper harvested rib bed.

**Figure 3.**
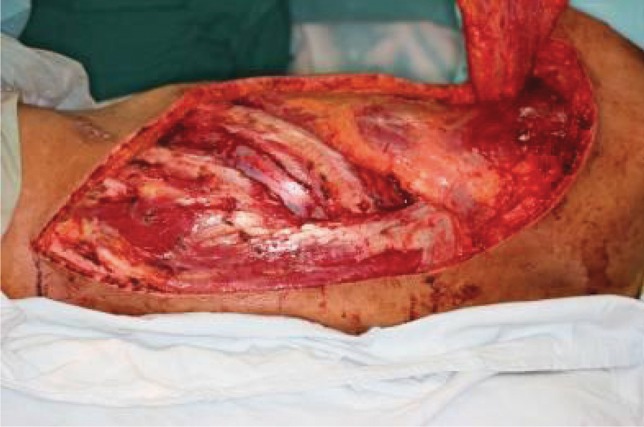
The perforators of the intercostals which in this flap are reversely nourishing the rib.

**Figure 4.**
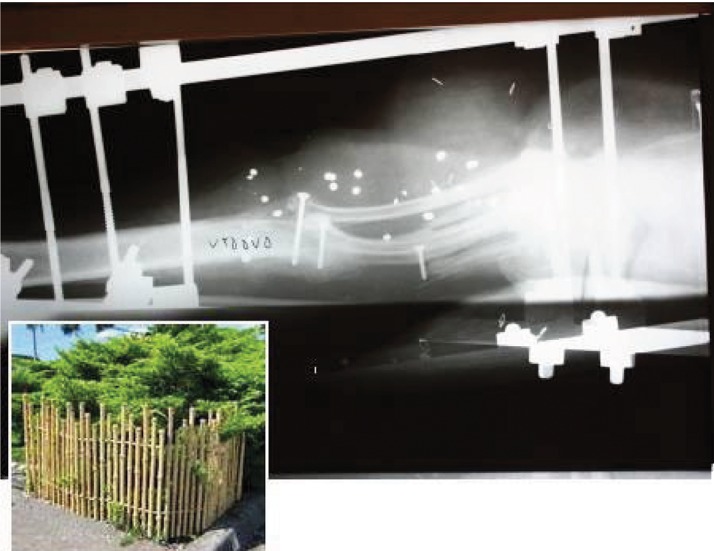
Four struts made out of two ribs, two short ones fill the defect and two long ones to bridge the gap and the ribs are fixed by titanium screws; a picture of “picket fence” is inserted at the lower border of the picture for similarity.

**Figure 5.**
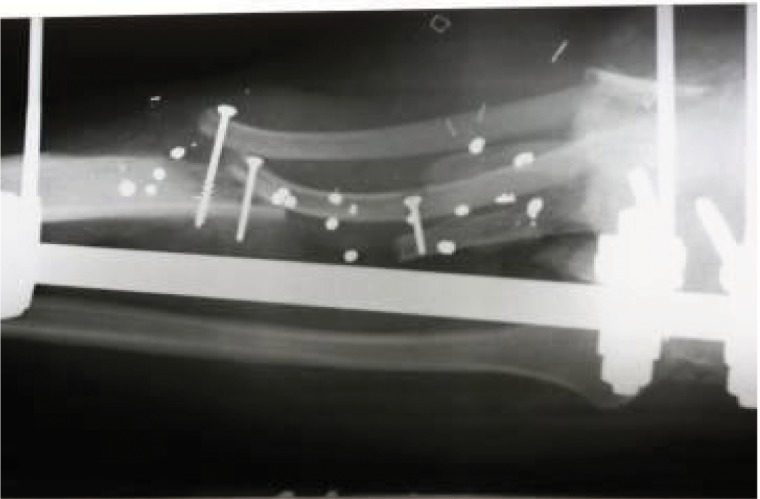
The result at six months; healing is complete but hypertrophy of the ribs is not yet seen.

**Figure 6.**
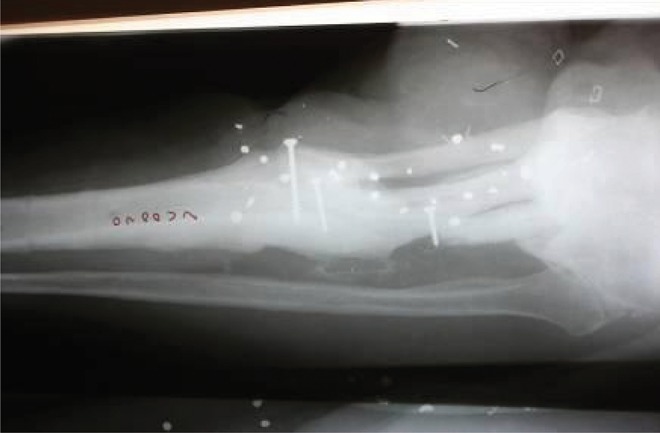
After nine months hypertrophy is progressing.

**Figure 7.**
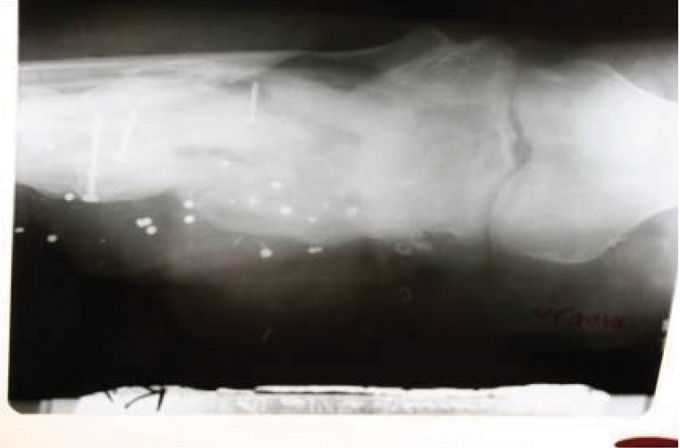
Three years postoperative, hypertrophy of the ribs grafted to the upper tibia can be seen.

From 2001 to 2010 seven patients with severe bone and soft tissue defects of extremities were seen at our clinic. They had upper tibia defects in which the “picket fence” reconstruction was used. The combo flap is elevated with one or two ribs (every other rib should be harvested to prevent collapse of the thoracic cage). Utmost care is taken to elevate the intercostal vessels with a rim of intercostal muscle and the perforating branch going into the muscle (Figures 1-3). After elevating the rib/s the flap is transferred to the defect and according to the defect the rib/s is or are bisected with the vessels intact. The rib struts are inserted longitudinally side by side filling the defect hence the “picket fence” design (Figure 4). Fixation of the bone is very important and either internal or external fixation or combinations of both are used. The use of LCP titanium plates is favored . After healing of the wound and bone healing gradual weight bearing is begun and the ribs hypertrophy with time and weight bearing. The result of a representative case is shown (Figures 5-7).

### 3.1. Case Report

**Figure 8.**
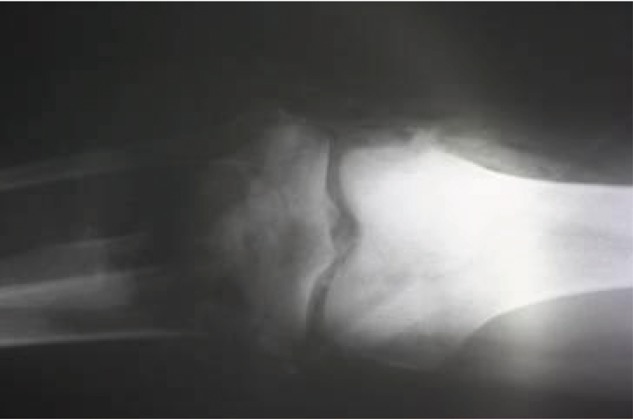
X-ray of the patient at the time of admission.

**Figure 9.**
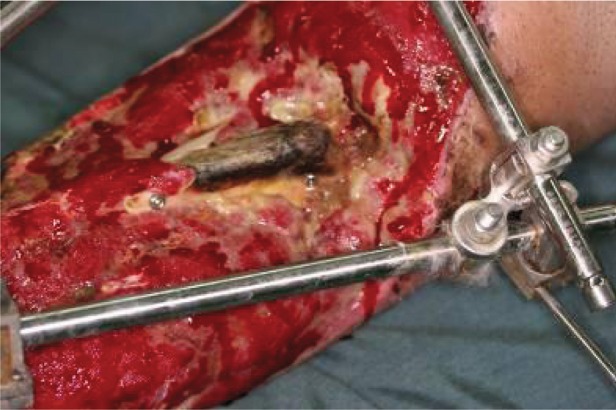
The leg 12 days after the accident, showing good granulation tissue and exposed tibia; at the first hospital they had fixed the tibia with two screws.

**Figure 10.**
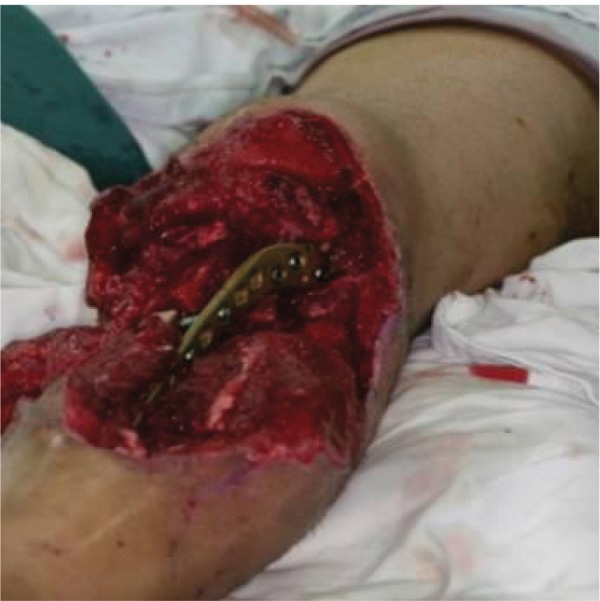
Fracture fixed by a titanium LCP plate; the necrotic bone was completely removed.

**Figure 11.**
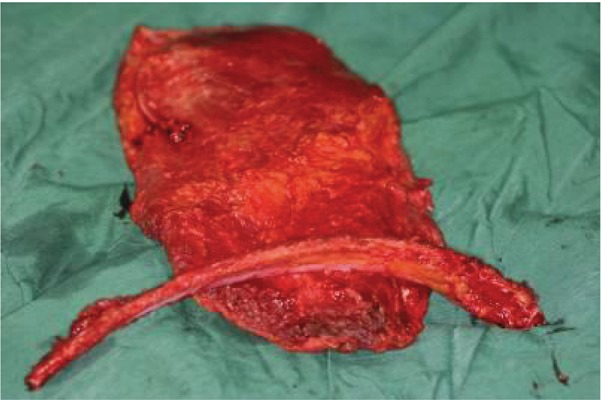
RLD muscluocutaneus flap with one rib harvested

**Figure 12.**
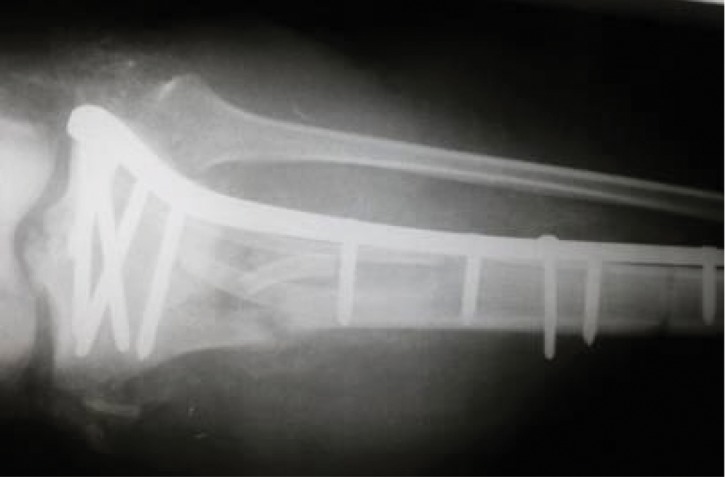
X-ray of the tibia with the rib trisected and used to fill the bone defect.

**Figure 13.**
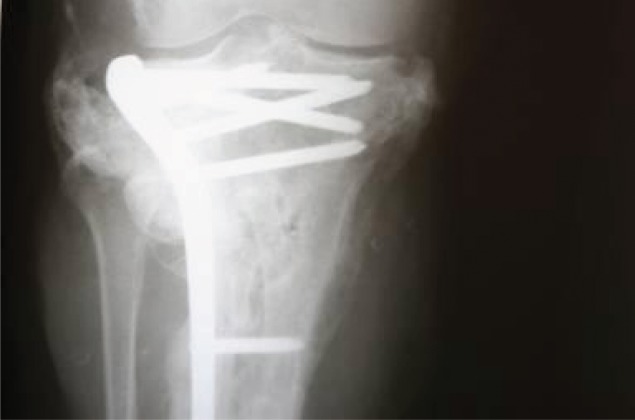
X-ray at 16 months; complete bony union and hypertrophy of the rib is noted

**Figure 14.**
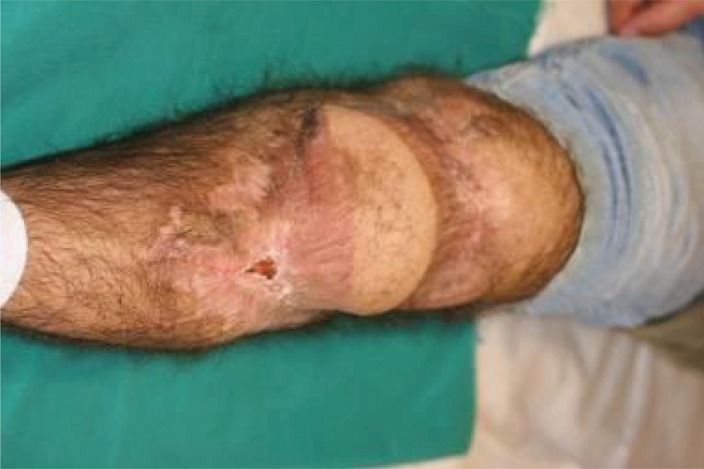
The wound completely healed and leg in extension

A 20 year-old patient 3 weeks after a car accident had a type 3b Gustilo fracture and at the first hospital the wound had been debrided and an external fixator applied. At the time of admission in our clinic the wound was clean but a 12 cm tibia defect with necrosis and a large soft tissue defect at the upper tibia with destruction of the anterior and anterolateral leg muscles was observed. The patient declined amputation and requested salvage of the leg. RLD muscle flap with one rib was harvested and the rib was trisected forming 3 struts. The flap was set in place and anastomosis of the vascular pedicle was to the popliteal vessels. The postoperative course was uneventful and after three months assisted weight bearing was begin and the patient was able to bear weight fully after nine months. The X-ray at nine months shows considerable hypertrophy of the bones (Figures 8-14).

## 4. Results

Complete flap survival was 100%. One flap had compromised circulation and underwent exploration. Although arterial obstruction was revised, partial necrosis of the skin paddle could not be avoided which was skin grafted at a later date. One additional flap also required revision as a result of venous congestion; it was totally salvaged. Superficial wound infection occurred in three patients, which was managed conservatively. The rate of primary bone union was 100%. At two year follow up only one case of persistent draining sinus was observed which was treated by curettage and removal of a sequestrum. The criterion used for bone graft hypertrophy was an increase in bone diameter greater than 20 percent. Gradual bony hypertrophy occurred in all the transferred rib flaps. The average hypertrophy rate was 148.6 percent. Stress fractures occurred in one patient which was located at the shaft of one of the rib grafts. The stress fracture was treated with a cast and external skeletal fixation. Four patients had anterolateral compartment muscle loss that required tendon transfers or ankle fusion to provide a plantigrade foot. Three donor sites had morbidity; these included pneumothorax (one patient), chest wall deformities (one patient), and chronic chest pain (1 patient) Table 1.

**Table 1.** Data of all Seven Patients Treated

Age (yr)	Cause of injury	Extremity	Ribs Harvested	Complications
4	RTA ^a^	Lower Extremity	Two ribs	Wound infection
22	RTA	Lower Extremity	One rib	-
25	RTA	Lower Extremity	One rib	Wound infection
47	RTA	Lower Extremity	Two ribs	Chest wall deformity
20	RTA	Lower Extremity	Two ribs	-
18	RTA	Lower Extremity	Two ribs	Chronic chest painpneumothorax
28	RTA	Lower Extremity	One rib	-

^a^ Abbreviation: RTA; Road Traffic Accident

## 5. Discussion

Vascularized fibular osteoseptocutaneous flaps are generally recommended as the first choice donor tissue for reconstruction of segmental long bone defects because the fibular flap has several advantages (12). When the fibular flap is not available however, the iliac osteocutaneous flap and serratus anterior rib flap become useful alternative donor sites .The LD muscle harvest has become a routine microsurgery procedure; adding the ribs to this flap is a bit difficult and needs meticulous care to preserve the perforator branches of intercostal arteries to the muscle and then these vessels act as the reverse flow to the rib when the ribs are elevated with the intercostal pedicle. The advantages of this flap are coverage of a large volume bone defect with simultaneous soft tissue coverage in one setting. The rib hypertrophies to the degree that it can bear weight and there is no need for any other bone graft. The previous studies have not reported the bisecting of the rib and bone filling as a one stage procedure for upper tibia defects. The LD-rib flap has both the long pedicle to bypass the zone of injury and also the volume of bone transferred is much larger than the fibula; the “Picket Fence” design uses four to six struts of vascularized rib; and last but not least the LD muscle is the largest muscle in the body and can cover large soft tissue defects. There is no need to harvest serratus muscle; LD is enough to vascularize the harvested ribs. From the above arguments it seems that LD-rib flap with “picket Fence” design is the best choice for upper tibia soft and hard tissue defects.

Transferred bone hypertrophy is a time-related phenomenon, and there are various factors involved in the speed and extent of bone graft hypertrophy. In this series, increased rib-bone hypertrophy occurred in all transferred LD-rib flaps. Patients should be encouraged to pursue early partial weight bearing with protection, thus exposing the bone grafts to stress to promote bone union, remodeling, and hypertrophy (13). The prevalence of stress fracture rates was 12.5 percent (one of 7 flaps). The stress fractures occur when patients fall, run, or are getting out of a bathtub without wearing the protective brace. Therefore, patient activity and brace protection are critical factors in postoperative care of the bone grafts (14, 15). All the patients were able to walk independently after two years. Limitations of the vascularized LD-rib chimera bone flaps to lower extremity reconstruction are as follows: 1) ribs are curved rather than straight. 2) It has limited cross-sectional area to withstand body weight load. For lower extremity reconstruction, one rib graft is too weak to support body weight. Theoretically, two or three sections of ribs should be used to provide more strength and support axial weight bearing.3) The ribs are membranous bone and have less mechanical strength that the cortical straight fibula. 4) The overall bone union and bone hypertrophy takes a long time, which affects the patient’s quality of daily life and the time before returning to work. 5) The rate of overall recipient and donor-site complications are higher than the other vascularized bone transfers. In particular, the common complication that develops in elevating this flap is pleural injury. This is another reason why clinical application of vascularized rib grafts has not been done extensively (16). The “picket fence” design presented in this article overcomes the problem of the “curvature” of the ribs and by placing the ribs side by side we can fill large upper tibia defects with vascularized bone ; it is indicated in a select group of patients. Type 3b and 3c Gustilo continue to be challenging for surgeons. The appropriate treatment of these wounds has been the subject of much research over time and the goals remain the same: early irrigation and debridement followed by early soft-tissue coverage (12). This study demonstrates a significant shift in practice, i.e. using a complex free flap for the primary wound closure. The LD-rib free flap seems to be the best choice in large volume soft and hard tissue defects of the lower extremity; the “PicketFence” rib design for upper tibia defects can be an addendum to the armamentarium of the reconstructive surgeon.

## References

[1] Buncke HJ, Furnas DW, Gordon L, Achauer BM (1977). Free osteocutaneous flap from a rib to the tibia.. Plast Reconstr Surg..

[2] Tu YK, Yen CY, Yeh WL, Wang IC, Wang KC, Ueng WN (2001). Reconstruction of posttraumatic long bone defect with free vascularized bone graft: good outcome in 48 patients with 6 years' follow-up.. Acta Orthop Scand..

[3] Ueba Y, Fujikawa S (1983). Nine years follow up of a free vascularized fibular graft in neurofibromatosis - a case report and literature review.. Jpn J Orthop Trauma Surg..

[4] Dunn R, Watson S (2001). Why climb a ladder when you can take the elevator?. Plast Reconstr Surg..

[5] Hallock GG (1997). Permutations of combined free flaps using the subscapular system.. J Reconstr Microsurg..

[6] Bennett N, Choudhary S (2000). Why climb a ladder when you can take the elevator?. Plast Reconstr Surg..

[7] Harii K, Yamada A, Ishihara K, Miki Y, Itoh M (1982). A free transfer of both latissimus dorsi and serratus anterior flaps with thoracodorsal vessel anastomoses.. Plast Reconstr Surg..

[8] Moscona RA, Ullmann Y, Hirshowitz B (1988). Free composite serratus anterior muscle--rib flap for reconstruction of severely damaged foot.. Ann Plast Surg..

[9] Georgescu AV, Ivan O (2003). Serratus anterior-rib free flap in limb bone reconstruction.. Microsurgery..

[10] Hui KC, Zhang F, Lineaweaver WC, Moon W, Buncke GM, Buncke HJ (1999). Serratus anterior-rib composite flap: anatomic studies and clinical application to hand reconstruction.. Ann Plast Surg..

[11] Derby LD, Bartlett SP, Low DW (1997). Serratus anterior free-tissue transfer: harvest-related morbidity in 34 consecutive cases and a review of the literature.. J Reconstr Microsurg..

[12] Nusbickel FR, Dell PC, McAndrew MP, Moore MM Vascularized autografts for reconstruction of skeletal defects following lower extremity trauma. A review.. Clin Orthop Relat Res..

[13] Urken ML, Weinberg H, Vickery C, Buchbinder D, Lawson W, Biller HF (1991). Oromandibular reconstruction using microvascular composite free flaps. Report of 71 cases and a new classification scheme for bony, soft-tissue, and neurologic defects.. Arch Otolaryngol Head Neck Surg..

[14] Arai K, Toh S, Tsubo K, Nishikawa S, Narita S, Miura H (2002). Complications of vascularized fibula graft for reconstruction of long bones.. Plast Reconstr Surg..

[15] Kasashima T, Minami A, Kutsumi K (1998). Late fracture of vascularized fibular grafts.. Microsurgery..

[16] Lin CH, Wei FC, Chen HC, Chuang DC (1999). Outcome comparison in traumatic lower-extremity reconstruction by using various composite vascularized bone transplantation.. Plast Reconstr Surg..

